# Association between coping strategies and professional quality of life in nurses and physicians during COVID‐19: A cross‐sectional study

**DOI:** 10.1111/jonm.13845

**Published:** 2022-10-26

**Authors:** Jessica Graziella Calegari, Selena Russo, Michela Luciani, Maria Grazia Strepparava, Stefania Di Mauro, Davide Ausili

**Affiliations:** ^1^ Department of Medicine and Surgery University of Milano‐Bicocca Monza Italy; ^2^ Healthcare Professions Department Fondazione IRCCS Ca' Granda Ospedale Maggiore Policlinico Milan Italy

**Keywords:** coping, COVID‐19, nurses, physicians, professional quality of life

## Abstract

**Aim:**

To explore the associations between coping strategies (social support, avoidance strategies, positive attitude, problem orientation, and transcendent orientation) and professional quality of life (compassion satisfaction, burnout, and secondary traumatic stress) of nurses and physicians during COVID‐19.

**Background:**

Little is known about the association between the way health care workers cope with stress and their professional quality of life during the unusual circumstances that the COVID‐19 pandemic imposed.

**Methods:**

A single‐centre cross‐sectional observational study was conducted with health care professionals (*n* = 143). The Professional Quality of Life scale Version 5 and the Italian Version of the Coping Orientations to the Problems Experienced measured the professional quality of life and coping strategies, respectively.

**Results:**

Avoidance, problem orientation and social support coping worsened professional quality of life, whereas a positive attitude improved it.

**Conclusions:**

This study on the relationship between coping strategies and the professional quality of life during health emergencies like the COVID‐19 pandemic can inform interventions aiming to foster functional coping strategies in health care personnel to sustain their professional quality of life.

**Implications for Nursing Management:**

Identifying people at greater risk of burnout and secondary traumatic stress can guide tailored interventions to improve health care workers' wellbeing. Increased professional quality of life might turn in improved quality of care and reduced absenteeism and intention to leave.

## 1 INTRODUCTION

The COVID‐19 (Coronavirus disease) pandemic changed people's lives due to mass restrictions, social isolation and economic recession (Xiong et al., 2020). This situation has led to an increase of mental and psychological disorders in the global population, which developed anxiety and depressive symptoms (Luo et al., 2020; Marvaldi et al., 2021). The worst impact on mental health was found in health care professionals who were exposed to additional potentially traumatic or stressful factors due to COVID‐19 psychosocial strain (Buselli et al., 2020; Magnavita et al., 2022), post‐traumatic stress symptoms, witnessing numerous deaths and high workloads (Marvaldi et al., 2021).

Worse mental health negatively affects the professional quality of life (An et al., 2020; Suryavanshi et al., 2020). The professional quality of life is the quality of life perceived in relation to work and investigates both the positive and negative aspects of helping others (Kim et al., 2015). In literature, multiple tools are used to measure the professional quality of life among health professionals (Kandula & Wake, 2021). According to our chosen framework (Stamm, 2010), the professional quality of life of health care professionals accounts for the positive and negative aspects: compassion fatigue and compassion satisfaction. Compassion fatigue has two components: burnout and secondary traumatic stress. Burnout comprises emotional exhaustion, depersonalization, a negative attitude, a lack of personal fulfilment and frustration, whereas secondary traumatic stress is the negative feeling driven by fear and work‐related trauma (Hinderer et al., 2014; Hooper et al., 2010; Hunsaker et al., 2015; Peters, 2018). Compassion satisfaction is a protective mechanism that stems from the satisfaction of helping others, from fulfilment and from reward because of caring for patients (Hinderer et al., 2014; Hooper et al., 2010).

Compassion fatigue (secondary traumatic stress and burnout) was found to be high in professionals who worked closely with COVID‐19 patients (Buselli et al., 2020; Galanis et al., 2021; Orrù et al., 2021; Ortega‐Galán et al., 2020; Ruiz‐Fernández et al., 2020; Trumello et al., 2020). Nevertheless, compassion satisfaction of frontline workers, who are directly responsible for COVID‐19 patients, was equal to or higher than that of those working in other wards or contexts (Buselli et al., 2020; Ortega‐Galán et al., 2020; Trumello et al., 2020). To deal with the negative effects caused by COVID‐19, coping could play a key role to improve the professional quality of life.

Coping is a complex and multidimensional process that has been defined as thoughts and behaviours used to manage the internal and external demands of situations that are appraised as stressful (Folkman & Moskowitz, 2004), being one of the main defences to protect mental health (Dehghani et al., 2018; Huang et al., 2020). Coping, according to our chosen conceptualization, includes five conceptual domains: social support (seeking understanding, seeking information and emotional release), avoidance strategies (denial, humour, use of drug/alcohol, behavioural disengagement and mental detachment), a positive attitude (acceptance, containment and positive reinterpretation), problem orientation (suppression, planning and activity) and transcendent orientation (spirituality and religion) (Foà et al., 2015; Huang et al., 2020; Sica et al., 2008).

During the COVID‐19 pandemic, a positive attitude in nurses and physicians towards stressful situations was a strong protective factor for stress, whereas seeking social support and avoidance‐based strategies were risk factors (Babore et al., 2020; Cai et al., 2020). However, other studies have shown that avoidance strategies like emotional disengagement significantly reduced psychological distress and post‐traumatic stress disorder (Labrague, 2021). The professional quality of life was not assessed in these studies, and the association between coping strategies and the professional quality of life in nurses and physicians during COVID‐19 is currently unknown. Some previous studies, conducted before COVID‐19, have investigated the association between quality of life and coping using different tools to measure the two concepts. They showed that coping strategies like problem orientation and social support could enhance compassion satisfaction, whereas avoidance‐based strategies could increase burnout (Al Barmawi et al., 2019; Portero de la Cruz et al., 2020). However, knowing the relationship between coping and the professional quality of life during COVID‐19 can inform and design specific interventions to promote effective coping strategies among professionals during emergencies that extend over time. This could potentially improve the professional quality of life of frontline nurses and physicians and prevent burnout and damaging mental health effects. Therefore, this study aimed to explore the associations between coping strategies (social support, avoidance strategies, positive attitude, problem orientation and transcendent orientation) and the professional quality of life (compassion satisfaction, burnout and secondary traumatic stress) in nurses and physicians during COVID‐19.

## 2 METHODS

This study uses a single‐centre cross‐sectional design. The study was conducted in a general public hospital in a large metropolitan city in the north of Italy. The hospital performs around 46,000 hospitalizations and 3 million outpatient services per year.

### 2.1 Participants and procedures

Between April and May 2021, all the 185 health care professionals working in the Emergency department, Pulmonology department, and Haematology and Bone Marrow Transplant Centre were invited to participate in a cross‐sectional online survey by invitation on their institutional email addresses. One author of the study (JGC) personally contacted nurses and physician managers of the three selected wards. The managers then sent all the nurses and physicians working in these three wards (*n* = 185) the email containing the presentation of the study and the survey. The managers also sent an email reminder every 15 days for a total of four emails. The three departments included three different COVID‐19 patient status conditions: only COVID‐19 positive (Pulmonology department), only COVID‐19 negative (Haematology and Bone Marrow Transplant Centre) and both COVID‐19 positive and COVID‐19 negative (Emergency department).

Inclusion criteria were being a nurse or a physician and having been working at the bedside for at least 6 months before the survey administration. No exclusion criteria were applied. Formal consent was obtained from each participant via the survey, and mandatory screening questions at the beginning of the survey assessed participants' eligibility. The questionnaires were anonymous, and no compensation for participation was provided.

### 2.2 Measures

Participants' sociodemographic characteristics were collected by a self‐report questionnaire, including gender, age, marital status, children, professional seniority, unit and COVID‐19 infection status.

The professional quality of life was measured with the Italian version of the Professional Quality of Life scale Version 5 (ProQOL‐5) (Palestini et al., 2009; Stamm, 2010). The ProQOL‐5 is a 30‐item self‐report questionnaire measuring three aspects of the professional quality of life. The positive aspect of ProQOL‐5 is *compassion satisfaction* and represents the pleasure derived from helping others through work. *Burnout* is the negative effect of caring that shows as exhaustion, frustration, anger and depression related to work and usually has a gradual onset. *Secondary traumatic stress* is the other negative aspect of ProQOL and is about feeling fear in relation to work‐related primary or secondary trauma. The compassion satisfaction subscale encompasses the positive aspects of helping others who have experienced suffering, whereas the burnout and the secondary traumatic stress subscales assess its negative consequences (*compassion fatigue*). Each subscale is unique and counts 10 items asking to indicate how frequently participants experienced a set of emotions or events in the previous 30 days to be rated on a 5‐point Likert scale ranging from 0 = *never* to 5 = *very often*. Higher scores on compassion satisfaction indicate that respondents are experiencing better satisfaction with their ability to provide care. Higher scores on burnout and secondary traumatic stress indicate that the respondents are at higher risk of experiencing symptoms of burnout and secondary traumatic stress, respectively. Validity was calculated from data from over 200,000 participants across the globe, and the ProQOL‐5 was found to be valid and reliable in several contexts and accounts for sociodemographic differences (Stamm, 2010). In Stamm (2010), the Cronbach's alpha was .88 for compassion satisfaction, .75 for burnout and .81 for secondary traumatic stress. In this study, we confirmed good reliability for each ProQOL‐5 subscale: burnout (α = .779), secondary traumatic stress (α = .819) and compassion satisfaction (α = .904) scales.

Participants' trait coping strategies, that is, the usual way people cope with stress, were assessed with the Italian Version of the Coping Orientations to the Problems Experienced (COPE‐NVI) (Carver et al., 1989; Foà et al., 2015). COPE‐NVI is a multidimensional inventory comprising 60 items evaluating how often the subject uses a set of particular coping processes in difficult or stressful situations and is rated on a 4‐point scale ranging from 1 = *I usually do not do this at all* to 4 = *I usually do this a lot*. The instrument includes five subscales corresponding to five different coping styles: *social support*, *avoidance strategies*, *positive attitude*, *problem orientation* and *transcendent orientation*. Social support is about seeking moral support from friends and relatives, trying to ask other advice on what to do, looking for the understanding of someone and solidarity. Avoidance strategies concern the abandonment of any attempt to act, the tendency to distract with alcohol and substances and the refusal to believe what happened. Positive attitude strategies involve learning to live with the problem, accepting the reality of the facts and looking for the positive side of events as something constructive. Problem orientation is about focussing on the problem and avoiding what may interfere with efforts to address the problem. Finally, transcendent orientation concerns praying and placing one's hope in God and trying to find comfort in religion. A higher score on a particular subscale indicates greater use of that specific coping strategy. COPE‐NVI has shown good psychometric properties (Babore et al., 2020), measuring reliably social support (α = .91), avoidance (α = .70), a positive attitude (α = .76), problem orientation (α = .83) and transcendent orientation (α = .85) (Sica et al., 2008). The Cronbach's alpha of factors in our study ranged from .78 to .86.

### 2.3 Statistical method

Sociodemographic characteristics were described by means, standard deviations and frequency distribution in the overall sample and by profession (physicians vs. nurses). Chi‐square test, z‐score test for proportions and unpaired *t* test or Mann–Whitney U test, when assumptions of normality were violated, were used to compare subgroups and explore possible gender differences. Effect size was measured by Cohen's *d*. Bivariate analyses were carried out to explore the association between the investigated variables. A set of one‐way analyses of variance was used to explore differences in professional quality of life dimensions between physicians and nurses working in the three different settings described above (only COVID‐19 positive, only COVID‐19 negative and both COVID‐19 positive and negative patients). Finally, multiple linear regression models were used to assess associations between coping strategies and professional quality of life dimensions. Model selection included major sociodemographic and professional factors such as gender, professional role and seniority, and those variables were found to have a significant relationship (*p* < .1) in bivariate analysis. We then used an iterative backward selection process examining *p* values to derive a parsimonious model (statistical significance was set at *p* < .05). All data analyses were performed using the statistical software package SPSS 27.0.

## 3 RESULTS

A total of 143 health care professionals consented to participate and completed the survey (response rate: 77.3%). Participants' demographic characteristics, professional information and COVID‐19‐related information are presented in Table 1. Overall, around 60% of participants were female, 40% had children and slightly less than 70% were married or in a de facto relationship. About 50% reported seniority up to 10 years, and nurses represented 66% of the study participants. Nurses were slightly older than physicians (χ^2^[3] = 8.244 *p* = .041). Less than 40% of participants reported being COVID‐19 positive at some point. Coping strategies' and professional quality of life dimensions' values by professional role are reported in Table 1. Nurses disclosed statistically significant lower levels of burnout and secondary traumatic stress compared with physicians.

**TABLE 1:** Descriptive sociodemographic characteristics, professional quality of life, professional information and health‐related features

	Total (*n* = 143)	Physicians (*n* = 48)	Nurses (*n* = 95)	*p* value
Gender				
Female (%)	89 (62.2%)	30 (62.5%)	59 (62.1%)	*z* = .046, ns
Age				χ^2^ (3) = 8.244 *p* = .041
20–29	24 (16.8%)	3 (6.3%)	21 (22.1%)	
30–39	83 (58%)	34 (70.8%)	49 (51.6%)	
40–49	21 (14.7%)	8 (16.7%)	13 (13.7%)	
50–59	15 (10.5%)	3 (6.3%)	12 (12.6%)	
**Marital status**				
Single	39 (27.3%)	15 (31.3%)	24 (25.3%)	χ^2^ (5) = 1.423 *p* = .922
Married	43 (30.1%)	14 (39.2%)	29 (30.5%)	
De facto	54 (37.8%)	17 (35.4%)	37 (38.9%)	
Divorced	2 (1.4%)	1 (2.1%)	1 (1.1%)	
Engaged	4 (2.8%)	1 (2.1%)	3 (3.2%)	
Widow(er)	1 (.7%)	‐	1 (1.1%)	
**Children—yes**	58 (40.6%)	17 (35.4%)	41 (43.2%)	*z* = −.890, ns
**Professional seniority**				
<10	79 (55.2%)	29 (60.4%)	50 (52.7%)	χ^2^ (3) = 6.222 *p* = .101
10–19	44 (30.8%)	17 (35.4%)	27 (28.4%)	
20–29	14 (9.8%)	2 (4.2%)	12 (12.6%)	
≥30	6 (4.2%)	‐	6 (6.3%)	
**Currently working in an unit that mainly manages patients:**				
Covid‐19−	28 (19.6%)	12 (25%)	16 (16.8%)	χ^2^ (2) = 2.388 *p* = .303
Covid‐19+	34 (23.8%)	13 (27.1%)	21 (22.1%)	
Both	81 (56.6%)	23 (47.9%)	58 (61.1%)	
**Ever resulted COVID‐19 positive**				
Yes	53 (37.1%)	13 (27.1%)	40 (42.1%)	*z* = −1.756, ns
**Professional quality of life**				
Compassion satisfaction	36.1 ± 7	35.6 ± 6.3	36.4 ± 7.4	*t*(141) = −.620, *p* = .536 *CI*_95_–3.25, 1.69, *d* = −.11
Burnout	25 ± 6.4	26.6 ± 5.7	24.3 ± 6.5	*t*(141) = 2.194, *p* = .030 *CI*_95_ .24, 4.63, *d* = −.39
Secondary traumatic stress	21 ± 6.8	22.6 ± 6.9	20.2 ± 6.6	*t*(141) = 2.039, *p* = .043 *CI*_95_ .07, 4.75, *d* = −.361
**Coping strategies**				
Social support	27.3 ± 6.9	28.8 ± 7.3	26.5 ± 6.7	*t*(141) = 1.854, *p* = .066 *CI*_95−_.15, 4.66, *d* = .33
Avoidance strategies	22.9 ± 4.9	23.1 ± 4.8	22.8 ± 4.9	*t*(141) = .351, *p* = .726 *CI*_95_–1.41, 2.02, *d* = .06
Positive attitude	31.5 ± 5.4	32.1 ± 4.5	31.2 ± 5.9	*t*(141) = .938, *p* = .350 *CI*_95_–1.0, 2.81, *d* = .17
Problem orientation	27.7 ± 5.4	28.8 ± 4	27.2 ± 6	*t*(141) = 1.655, *p* = .100 *CI*_95−_.31, 3.471, *d* = .29
Transcendent orientation	20.1 ± 3.9	19.4 ± 4.4	20.5 ± 3.6	*t*(141) = −1.545, *p* = .125 *CI*_95_–2.43, .29, *d* = −.27

Abbreviation: CI = confidence interval.

Further, Cohen's effect size values (*d* = .39 and *d* = .36, respectively) suggested a small effect. The mean values for compassion satisfaction were not significantly different for nurses compared with physicians. Compared to their male counterpart, female health care workers reported more often availing social support and turning to religion to manage stressful situations. No gender differences emerged in the dimensions (Table 2).

**TABLE 2:** Coping strategies and professional quality of life dimensions by gender

	Total (*n* = 143)	Female (*n* = 89)	Male (*n* = 54)	*p* value
**Professional quality of life**				
Compassion satisfaction	36.1 ± 7	35.5 ± 7.1	37.1 ± 6.8	*t*(141) = 1.319, *p* = .189 *CI*_95_–3.25, 1.69, *d* = .29
Burnout	25 ± 6.4	25.2 ± 6.5	24.8 ± 6.1	*t*(141) = −.393, *p* = .695 *CI*_95_–3.25, 1.69, *d* = .29
Secondary traumatic stress	21 ± 6.8	21.9 ± 7.1	19.6 ± 5.9	*t*(141) = −1.966, *p* = .051 *CI*_95_–3.25, 1.69, *d* = .29
**Coping strategies**				
Social support	27.3 ± 6.9	28.7 ± 7.1	24.9 ± 5.9	*t*(141) = −3.285, *p* = .001 *CI*_95_–5.73, −1.29, *d* = −.52
Avoidance strategies	22.9 ± 4.9	22.6 ± 4.4	23.3 ± 5.5	*t*(141) = .897, *p* = .371 *CI*_95−_.59, 2.71, *d* = .21
Positive attitude	31.5 ± 5.4	31.3 ± 5.6	31.7 ± 5.1	*t*(141) = .377, *p* = .706 *CI*_95_–1.62, 2.0, *d* = .03
Problem orientation	27.7 ± 5.4	27.3 ± 5.5	28.4 ± 5.3	*t*(141) = 1.249, *p* = .214 *CI*_95−_.41, 3.14, *d* = .25
Transcendent orientation	20.1 ± 3.9	21.1 ± 3.7	18.6 ± 3.7	*t*(141) = −3.818, *p* < .001 *CI*_95_–3.85, −1.32, *d* = −.68

Abbreviation: CI, confidence interval.

No statistically significant differences emerged between health care workers caring for the three COVID‐19 patient status condition groups (only COVID‐19 positive, only COVID‐19 negative and both COVID‐19 positive and COVID‐19 negative) for compassion satisfaction F(2, 140) = .300 *p* = .741; burnout F(2, 140) = .133 *p* = .875; and secondary traumatic stress F(2, 140) = .470 *p* = .626. The multiple regression model statistically significantly predicted compassion satisfaction, F(8, 134) = 8.57, *p* < .001, adj. R^2^ = .29. Professional seniority, a positive attitude and avoidance coping strategies added statistically significantly to the prediction, *p* < .05. Professional seniority, a positive attitude, problem orientation and avoidance coping strategies statistically significantly predict burnout F(8, 134) = 7.024, *p* < .001, adj. R^2^ = .253. Secondary traumatic stress was significantly associated with avoidance, problem orientation, social support and a positive attitude F(8, 134) = 7.866, *p* < .001, adj. R^2^ = .279. Regression coefficients and standard errors can be found in Table 3.

**TABLE 3:** Multiple linear regression analyses exploring the influence of coping strategies on professional quality of life dimensions

	B (SE)	β	95% CI for B		*p*	*R* ^*2*^	Δ*R* ^*2*^
			LL	UL			
**Professional quality of life—compassion satisfaction**							
Model						.338	.299
Constant	18.27 (5.04)		8.296	28.24	.000		
Gender	−2.19 (1.18)	−.151	.‐4.5	.131	.064		
Professional role	.994 (1.09)	.067	−1.153	3.142	.361		
Professional seniority	1.371 (.64)	.161	.107	2.63	.034		
Social support	.046 (.087)	.045	−.126	.218	.599		
Avoidance strategies	−.224 (.106)	−.155	−.434	−.014	.037		
Positive attitude	.665 (.140)	.514	.389	.942	.000		
Problem orientation	.003 (.149)	.003	−.291	.298	.982		
Transcendent orientation	.016 (.141)	.009	−.264	.296	.908		
**Professional quality of life—burnout**							
Model						.295	.253
Constant	26.058 (4.692)		16.778	35.338	.000		
Gender	2.142 (1.09)	.164	−.014	4.298	.052		
Professional role	−1.701 (1.01)	−.127	−3.7	.297	.095		
Professional seniority	−1.439 (.595)	−.188	−2.616	−.263	.017		
Social support	−.071 (.081)	−.078	−.231	.089	.381		
Avoidance strategies	.468 (.099)	.360	.272	.663	.000		
Positive attitude	−.599 (.130)	−.513	−.857	−.342	.000		
Problem orientation	.472 (.139)	.404	.198	.746	.001		
Transcendent orientation	−.115 (.132)	−.071	−.375	.146	.384		
**Professional quality of life—secondary traumatic stress**							
Model						.320	.279
Constant	.178 (4.91)		−9.526	9.882	.971		
Gender	2.136 (1.14)	.154	−.119	4.391	.063		
Professional role	−1.614 (1.057)	−.113	−3.704	.475	.129		
Professional seniority	−.461 (.622)	−.056	−1.691	.769	.460		
Social support	.171 (.085)	.175	.003	.339	.046		
Avoidance strategies	.591 (.103)	.427	.386	.795	.000		
Positive attitude	−.274 (.136)	−.220	−.543	−.004	.046		
Problem orientation	.307 (.145)	.247	.021	.594	.036		
Transcendent orientation	.137 (.138)	.079	−.136	.409	.323		

*Note*: In bolds *p* values < .05.

Abbreviations: B, beta; SE, standard error; CI, confidence interval; LL, lower limit; UL, upper limit.

## 4 DISCUSSION

The study aimed to explore the associations between coping strategies (social support, avoidance strategies, positive attitude, problem orientation and transcendent orientation) and the professional quality of life (compassion satisfaction, burnout and secondary traumatic stress) among nurses and physicians during COVID‐19. To the best of our knowledge, this is the first study to do so. We found that coping strategies used by health care professionals might affect compassion satisfaction and compassion fatigue (burnout and secondary traumatic stress) in the current pandemic. In particular, avoidance worsens the professional quality of life increasing burnout and secondary traumatic stress and reducing compassion satisfaction. Also using problem orientation and social support lead to a similar result, increasing burnout and secondary traumatic stress. Lastly, a positive attitude improved the professional quality of life, reducing burnout and secondary traumatic stress and improving compassion satisfaction. This is relevant because the professional quality of life could be improved by interventions focussed on enhancing functional coping strategies.

A positive attitude and avoidance coping strategies were positive and negative predictors of compassion satisfaction, respectively. This is coherent with previous research conducted before the COVID‐19 pandemic (El‐Shafei et al., 2018; Hinderer et al., 2014). It is possible that the positive reinterpretation of events and critical processing fostered greater psychological wellbeing (Babore et al., 2020; Cai et al., 2020; El‐Shafei et al., 2018), whereas the use of denial, humour, substance abuse and behavioural and mental disengagement affected stress and anxiety (Babore et al., 2020; El‐Shafei et al., 2018; Hinderer et al., 2014; Labrague, 2021; Savitsky et al., 2020).

Avoidance and problem orientation were positive predictors of compassion fatigue. Although the negative mechanism of avoidance on the professional quality of life is coherent with previous literature (Babore et al., 2020; El‐Shafei et al., 2018; Hinderer et al., 2014; Labrague, 2021; Savitsky et al., 2020), problem orientation has previously been positively associated with compassion satisfaction, therefore reducing compassion fatigue (Al Barmawi et al., 2019; El‐Shafei et al., 2018). It is possible that the unpredictability of the COVID‐19 pandemic affected problem orientation, making health care professionals feel inadequate to control the situation and their perception of problem‐solving abilities (Babore et al., 2020).

Social support was a positive predictor of secondary traumatic stress, a dimension of compassion fatigue. This is in contrast with previous literature, in which social support significantly reduced stress (Labrague, 2021; Luo et al., 2020; Sanghera et al., 2020; Spoorthy et al., 2020) but partly coherent with a recent study conducted during the pandemic that found an association between higher social support and higher stress (Babore et al., 2020). This negative role of social support could be explained by focussing on the definition of social support by the COPE‐NVI scale, which also includes information seeking. In the current pandemic, information was often conflicting and contradictory, there was fake and misleading news from various sources, including institutional ones and exposure to social media and COVID‐19 news caused anxiety and stress, increasing fears and producing cognitive overload from infodemic (Fan & Smith, 2021; Mohammed et al., 2021; Savitsky et al., 2020). Furthermore, society's reaction towards health care professionals was not always positive. After the first wave of support and recognition for the work and effort of health care professionals (Portero de la Cruz et al., 2020), there was a progressive decrease in this social recognition combined with the increase of a pandemic and vaccine denier movement (Giordani et al., 2021; Herrera‐Peco et al., 2021), and health care professionals were victims of insults and threats and were accused of propaganda, which might justify the role of society in secondary traumatic stress (Bagcchi, 2020; Chirico et al., 2022; Ford, 2021). Lastly, social distancing rules and fear of infecting loved ones might have increased work‐related fear and trauma (Babore et al., 2020).

Seniority was a positive predictor of compassion satisfaction and a negative predictor of burnout. Staff with more experience probably developed protective mechanisms to reduce stress and hence burnout and to increase job satisfaction, whereas less experienced professionals are more vulnerable to facing difficult situations and tend to develop more stress and anxiety in dealing with daily challenges in a constantly unstable work environment, coherently with results from previous studies (Galanis et al., 2021; Hunsaker et al., 2015; Kelly et al., 2015; Portero de la Cruz et al., 2020).

Burnout and compassion satisfaction overall values were moderate, whereas secondary traumatic stress was low in our sample, in line with the results identified in the pre‐pandemic era (Kelly et al., 2015). This is in contrast with previous studies that highlighted how health care professionals have recorded higher levels of stress, anxiety and depression as well as burnout and secondary traumatic stress during the COVID‐19 pandemic (Galanis et al., 2021; Li et al., 2021; Marvaldi et al., 2021; Pappa et al., 2020; Sanghera et al., 2020; Varghese et al., 2021; Vindegaard & Benros, 2020). This difference might be explained by the time during which our study was conducted. We collected data between April and May 2021, towards the end of the third COVID‐19 wave in Italy and in the middle of the first vaccination campaign. The protection against infection from SARS‐CoV‐2 by vaccines, the decrease in mortality rates and the lower number of hospitalizations may have helped in relieving stress and anxiety symptoms in health professionals, leading to lower secondary traumatic stress and burnout levels.

Finally, female health care professionals used significantly more transcendent orientation and social support than males. This is coherent with previous studies that have shown gender differences in coping abilities (Cai et al., 2020). In fact, women are more likely to develop social and personal mechanisms to cope with stress than men, and they are likely to perform coping strategies that can release emotional stress by searching for support from colleagues, family and friends (Cai et al., 2020; Huang et al., 2020; Sica et al., 2008; Xiong et al., 2020). Lastly, unlike what emerged from recent systematic reviews (Danet Danet, 2021; Pappa et al., 2020), who found higher levels of burnout, stress, anxiety and depression among nurses than other health care professionals, within our study, physicians reported more compassion fatigue than nurses. This result is surprising; it may be linked to the ability to deal with powerlessness and uncertainty and should be further investigated.

### 4.1 Limitations and strengths

Due to the lack of consensus on the concepts of coping and the professional quality of life, several instruments are used throughout the literature for both concepts. This could hinder the comparison of results among different theoretical conceptualizations. However, in our study, we used the ProQOL‐5 and COPE‐NVI scales, which are both widespread and validated instruments. The cross‐sectional nature of the study does not allow assuming causality, and it was a monocentric study conducted on a sample of 143 nurses and physicians. Furthermore, the level of coping and the professional quality of life were not known in this population before the pandemic.

Mixed methods longitudinal studies with bigger and more diverse samples are required to better understand the relationship between coping and the professional quality of life. Furthermore, multiple variables should be considered for their mediating of confounding effects. Nevertheless, this is the first study that evaluated the association between the COPE‐NVI and ProQOL‐5 scores in nurses and physicians during the pandemic and that did so with a rigorous methodology, so our findings can be considered robust and generalizable to similar populations.

## 5 CONCLUSIONS

Nurses and physicians reported moderate levels of burnout and compassion satisfaction. Furthermore, coping strategies used by health care professionals affected compassion satisfaction, burnout and secondary traumatic stress in the current pandemic. Further studies are needed to corroborate our data, also using a bigger and more diverse sample with different health care professionals and with longitudinal mixed methods designs. Furthermore, future studies should also consider the effect on the professional quality of life of other occupational stressors, such as horizontal and vertical violence against health care professionals, overworking and staffing.

## 6 IMPLICATION FOR NURSING MANAGEMENT

Knowing which coping strategies and sociodemographic characteristics affect the professional quality of life can help managers identify those people more at risk of burnout and secondary traumatic stress. In particular, in our results, avoidance, problem orientation and social support coping worsened the professional quality of life, whereas a positive attitude improved it. These results could help management to design specific interventions and proactive strategies to improve the workers wellbeing and to minimize the level of compassion fatigue and burnout. Future research should focus on understanding which interventions are better at increasing the professional quality of life.

## CONFLICTS OF INTEREST

The authors have no conflicts of interest to disclose in relation to this manuscript.

## ETHICS STATEMENT

The study was approved by the Ethical Committee of the IRCCS Ca' Granda Ospedale Maggiore Policlinico Milano (Approval number: 309_2021). Participants were informed about the study before accessing the questionnaire, and they could leave the questionnaire at any time during completion. Health care professionals were not compensated for their participation in the study, and participation was voluntary. Data were collected anonymously, and all participants gave their consent electronically before entering the questionnaire.

## Data Availability

The data that support the findings of this study are available on request from the corresponding author. The data are not publicly available due to privacy or ethical restrictions.
